# OSblca: A Web Server for Investigating Prognostic Biomarkers of Bladder Cancer Patients

**DOI:** 10.3389/fonc.2019.00466

**Published:** 2019-06-04

**Authors:** Guosen Zhang, Qiang Wang, Mengsi Yang, Quan Yuan, Yifang Dang, Xiaoxiao Sun, Yang An, Huan Dong, Longxiang Xie, Wan Zhu, Yunlong Wang, Xiangqian Guo

**Affiliations:** ^1^Cell Signal Transduction Laboratory, Department of Preventive Medicine, Bioinformatics Center, School of Basic Medical Sciences, School of Software, Institute of Biomedical Informatics, Henan University, Kaifeng, China; ^2^Department of Anesthesia, Stanford University, Stanford, CA, United States; ^3^Henan Bioengineering Research Center, Zhengzhou, China

**Keywords:** bladder cancer, prognostic biomarker analysis, web server, kaplan-meier curve, cox regression model

## Abstract

Bladder cancer (BC) is one of the most common malignant tumors in the urinary system. The discovery of prognostic biomarkers is still one of the major challenges to improve clinical treatment of BC patients. In order to assist biologists and clinicians in easily evaluating the prognostic potency of genes in BC patients, we developed a user-friendly Online consensus Survival tool for bladder cancer (OSblca), to analyze the prognostic value of genes. The OSblca includes gene expression profiles of 1,075 BC patients and their respective clinical follow-up information. The clinical follow-up data include overall survival (OS), disease specific survival (DSS), disease free interval (DFI), and progression free interval (PFI). To analyze the prognostic value of a gene, users only need to input the official gene symbol and then click the “Kaplan-Meier plot” button, and Kaplan-Meier curve with the hazard ratio, 95% confidence intervals and log-rank *P*-value are generated and graphically displayed on the website using default options. For advanced analysis, users could limit their analysis by confounding factors including data source, survival type, TNM stage, histological type, smoking history, gender, lymph invasion, and race, which are set up as optional parameters to meet the specific needs of different researchers. To test the performance of the web server, we have tested and validated its reliability using previously reported prognostic biomarkers, including *KPNA2, TP53*, and *MYC etc*., which had their prognostic values validated as reported in OSblca. In conclusion, OSblca is a useful tool to evaluate and discover novel prognostic biomarkers in BC. The web server can be accessed at http://bioinfo.henu.edu.cn/BLCA/BLCAList.jsp.

## Introduction

As one of the most common malignant tumors of the urinary system, bladder cancer (BC) is estimated to cause about 549,393 new cases and 199,922 deaths worldwide in 2018 (1). Based on the clinic-pathological features, BC could be classified into two types: non-muscle invasive tumor (NMIBC, 70–80% of BC patient) and muscle-invasive tumor (MIBC, 20–30% of BC patient) (2, 3). Due to the relatively high rate of local recurrence and metastasis in MIBC patients, the treatment outcome is still poor, and the survival rate is lower than that of NMIBC patients. Although NMIBC patients have better survival rates than MIBC, 30–50% of NMIBC patients experience cancer recurrence (4). One of the major challenges to improve clinical outcomes of BC patients is to screen novel biomarkers for diagnosis and prognosis (5).

In recent years, a large number of prognostic biomarkers including DNA markers and protein markers have been reported (6–8). Some of the prognostic biomarkers, especially the ones involved in biological processes, are useful to identify high-risk patients, and could be used to predict the prognosis and treatment response. However, few biomarkers have been translated into clinics due to the lack of independent validation (5, 9, 10). With the advance of high through-put technologies, more and more studies analyzed the gene expression of cancer samples and uploaded these data on public databases such as The Cancer Genome Atlas (TCGA, https://portal.gdc.cancer.gov/) and Gene Expression Omnibus (GEO, https://www.ncbi.nlm.nih.gov/geo/). These data offer opportunities for the biomarker discovery, validation, and clinical application (11, 12). Unfortunately, until now, this convenient online tool is still unavailable to clinicians and biologists to evaluate and verify the prognostic value of the genes of interests in different datasets for BC.

To solve this problem, we developed an online web server named OSblca, which consists of gene expression profiles and relative clinical information of 1,075 bladder cancer patients from seven independent cohorts collected from TCGA and GEO databases. This web server enables researchers and clinicians to analyze the prognostic value of a gene of interest and accelerates the development of prognostic biomarkers.

## Methods

### Datasets Collection

Gene expression profiles and clinical follow-up information of bladder cancer patients were collected from TCGA and GEO databases. For TCGA dataset, level-3 gene expression profiling data (HiSeqV2) and clinical information of BC samples were downloaded in April 2018. In order to collect the relative datasets from GEO, keywords including “bladder cancer,” “prognosis,” “survival,” and “gene expression” were used to search in GEO database. Next, manual checks of the availability of data of mRNA expression, clinical survival information and at least 50 patients were performed.

### Development of OSblca

**Figure 1:**
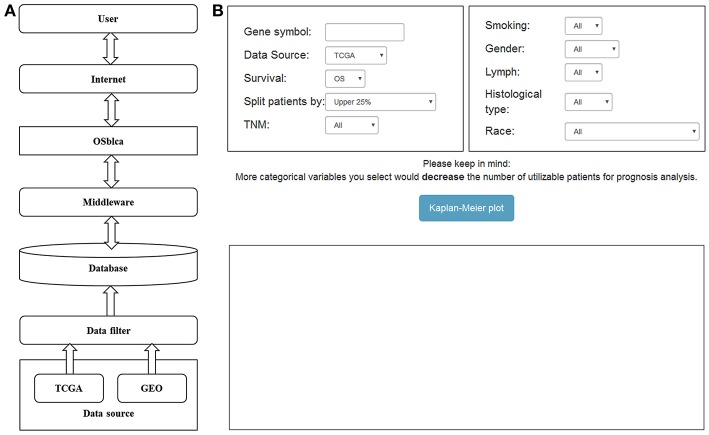
Diagram of web server architecture **(A)** and screenshot of OSblca main interface **(B)**.

The OSblca web server was developed by Java script, and hosted by Tomcat 7.0 on Windows 2008. The database system that stores the gene expression and clinical data was handled by SQL Server 2008. The R package “RODBC” is used as a middleware to connect R and SQL. The input of OSblca web server must be the official gene symbol from NCBI (https://www.ncbi.nlm.nih.gov/). The outputs include Kaplan Meier (KM) survival curves, Hazard ratio (HR with 95% confidence interval) and log-rank *P*-value that are produced by R package “survival” (https://CRAN.R-project.org/package=survival). A gene could be regarded as a potential prognostic biomarker for BC patients when the log-rank *P*-value is < 0.05. OSblca can be accessed at http://bioinfo.henu.edu.cn/BLCA/BLCAList.jsp. A web server architecture diagram is presented in Figure 1A. The screenshot of the web server interface and the result are shown in Figure 1B.

### Validation of Previously Published Prognostic Biomarkers in OSblca

In order to validate the performance of prognostic analysis in our web server, prognosis biomarkers for BC were searched in PubMed using the keywords “bladder cancer,” “survival,” “gene expression,” “biomarker,” and “prognosis.” The prognostic capabilities of these genes were evaluated in all cohorts, and all cutoff values in “splitting the patients” were tested in each cohort to get the best cutoff value.

## Results

### Clinical Characteristics of the Patients in OSblca

According to our criteria, in total 1,075 unique bladder cancer patients were collected from seven data sets including one TCGA cohort and six GEO cohorts. Survival information including overall survival (OS), disease specific survival (DSS), disease free interval (DFI), progression free interval (PFI) were gathered. No patient was lost to follow-up. Of the above, 935 patients have overall survival information, and the median overall survival time is 25.03 months. We also collected age, TNM stage, histological type, gender, smoking history, lymph invasion and race as confounding clinical factors. The average age is 68 ± 11. Distribution of TNM stages is as follows: stage I (*n* = 287, 29.64%), stage II (*n* = 233, 23.26%), stage III (*n* = 239, 23.85%), and stage IV (*n* = 209, 20.86%). The ratio of male to female of patients was close to 3:1. A summary of clinical properties for each dataset is presented in Table 1.

**Table 1:** Clinical characteristics of the BC patients collected in OSblca.

**Data source**	**Platform**	**Sample size**	**Age**	**No. of death**	**Media (OS)**	**Gender (% male)**	**Stage (%I/II/III/IV/NA^a^)**	**Never smokers (%)**	**Survival terms**
TCGA	Illumina HiSeqV2	407	69 ± 11	155	16.93	73.71	0.49/31.70/34.40/32.92/0.49	26.78	OS, DSS, DFI, PFI^*^
GSE13507	GPL6102	165	65 ± 12	69	36.57	–	48.48/15.76/11.52/10.30/13.94	–	OS
GSE19915	GPL3883/GPL5186	140		24	–	–	69.29/12.14/15.00/2.14/1.43	–	DSS
GSE31684	GPL570	93	69 ± 10	65	31.31	71.12	16.13/18.28/45.16/20.43/0.00	19.35	OS, DSS, DFI, PFI
GSE32548	GPL6947	130	70 ± 11	25	53.77	76.15	70.00/29.23/0.00/0.00/0.77	–	OS, DSS, DFI, PFI
GSE48075	GPL6947	73	69 ± 10	45	30.40	–	–	–	OS
GSE48276	GPL14951	67		31	34.10	80.60	2.99/8.96/25.37/53.73/8.95		OS, DSS
Total		1075	68 ± 11	414	25.03	74.46	29.64/23.26/23.85/20.86/3.39	11.81	

a NA, Not Available;

“–,” no data;

* *DFI and PFI were defined by (13)*.

### Survival Analysis of BC Patients Based on Clinical Characteristics

**Figure 2:**
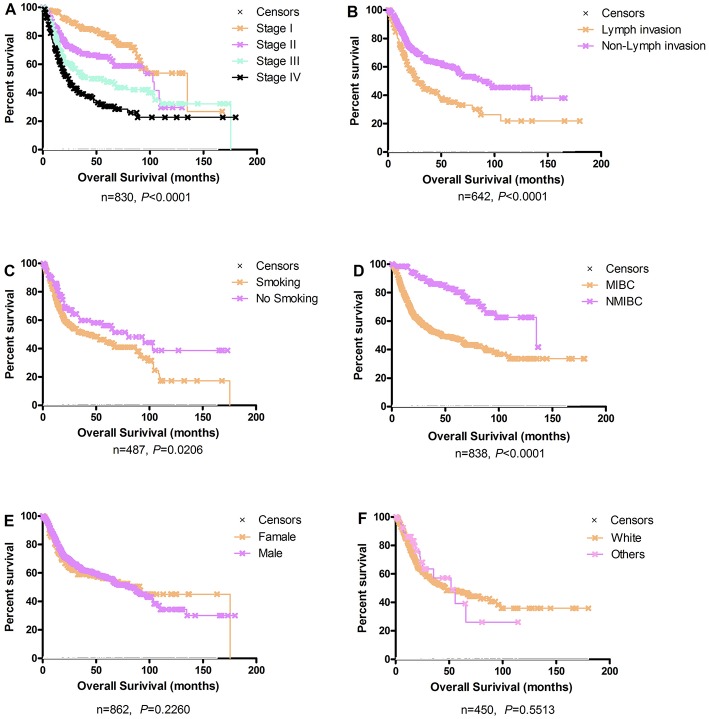
Survival analysis of clinical characteristics of the bladder cancer patients in OSblca. **(A)** TNM stage; **(B)** Lymph invasion; **(C)** Smoking history; **(D)** Histological type; **(E)** Gender; **(F)** Race.

The Kaplan-Meier plots for the bladder cancer patients in OSblca stratified by TNM stage, histological type, gender, smoking history, lymph invasion, and race are presented in Figure 2. In these 1,075 patients, TNM stage, smoking history, lymph invasion, and histological type were significantly associated with overall survival (*P* < 0.0001, *P* = 0.0206, *P* < 0.0001, and *P* < 0.0001, respectively), which were consistent with previously reports (14–16). Nevertheless, gender and race showed no significant association with overall survival (*P* = 0.2260 and *P* = 0.5513).

### Usage of OSblca

**Figure 3:**
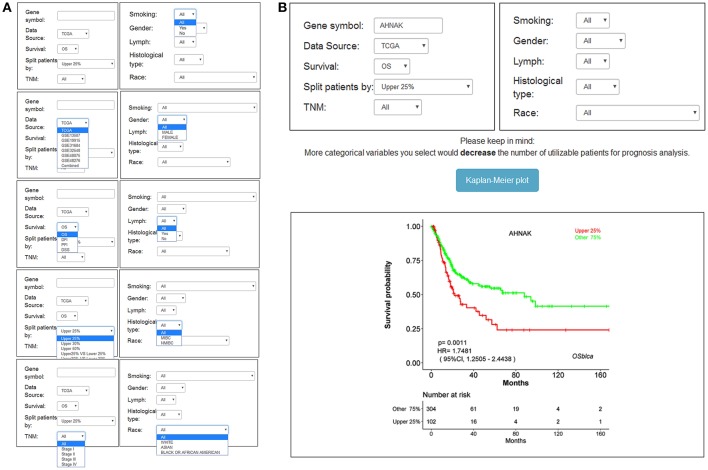
Input and output interface of OSblca. **(A)** The options of main input parameters and clinical factors of OSblca; **(B)** The output interface of OSblca.

The main function that OSblca provides is to evaluate and verify the prognostic value for a given gene. “Gene symbol,” “Data source,” “Survival,” and “Split patients” are set as the four main parameters. The input dialog box of “Gene symbol” is on the upper left of the OSblca page (Figure 3A). A red prompting message will show up when the input is not an official gene symbol. “Data source” provides eight options including independent analysis in one of seven cohorts and in a combined cohort consisting of all the BC patients from seven cohorts. The users can choose to evaluate the prognosis of a given gene in an individual cohort or in a combined cohort according to their needs. Under “Survival” option, four prognostic terms including OS, DSS, DFI, and PFI are provided. In the “Split patients” dialog box, user can select different thresholds of gene expression levels to divide patients into two subgroups for input gene. After then, by clicking the “Kaplan-Meier plot” button, OSblca server will take the request and return the analysis results, which are graphically displayed and presented with HR, 95% CI and log-rank *P*-value (Figure 3B).

In order to meet the specific needs, six confounding clinical factors including TNM stage, smoking history, gender, lymph, histological type, and race, were set as optional filter factors in the prognostic analysis. As showed in Figure 3A, each factor has 2–5 options for users to choose from.

### Validation of Previously Published BC Biomarkers

To test the reliability of prognosis prediction in our web server, we evaluated 21 prognostic biomarkers from 16 previously reported literatures in the OSblca web server, including *KPNA2, TP53*, and *MYC* (17–32). As shown in Table 2, 17 out of 21 (82%) previous reported prognostic biomarkers were showed to have significant prognostic potency in OSblca, while the remaining four previously reported prognostic biomarkers did not reach significance in OSblca. Among the 17 validated prognostic biomarkers, 11 genes showed significant prognostic abilities in the combined cohort.

**Table 2:** The validation of previous reported prognostic biomarkers in OSblca.

**Gene symbol**	**Detection level**	**Case**	**Independent validation**	**In reference**			**In OSblca**		
				***P*-value**	**HR**	**References**	***P*-value**	**HR**	**Cut off**
*KPNA2*	Protein	611	Yes	0.030	1.38	(17)	0.001^a^	1.42	Upper 25%
*HYAL1*	Protein	220	Yes	0.019	1.76	(18)	0.021^a^	1.29	Upper 25%
*TP53*	Protein	152	No	< 0.001	–	(19)	0.037^a^	0.77	Upper 25%
*MYC*	Protein	132	No	0.020	–	(20)	0.050^a^	1.25	Upper 25%
*RPS6*	Protein	132	No	< 0.010	–	(20)	0.007^a^	0.71	Upper 25%
*JMJD2A*	Protein	129	No	0.033	–	(21)	0.026^a^	0.63	Upper 25%
*MKi67*	Protein	115	No	< 0.050	–	(22)	0.021^a^	1.29	Upper 25%
*RRM1*	Protein	84	No	0.001	–	(23)	0.000^a^	1.68	Lower 25%
*MMP2*	mRNA	41	No	< 0.05	–	(25)	0.039^a^	1.26	Upper 25%
*CDH2*	mRNA	181	No	< 0.001	–	(26)	0.038^a^	1.26	Upper 25%
*PTGS2*	Protein	273	No	0.027	0.65	(24)	0.050^b^	0.72	Upper 25%
*CDH3*	mRNA	181	No	< 0.010	–	(26)	0.041^c^	2.30	Upper 25%
*MDM2*	Protein	84	No	< 0.050		(27)	0.045^d^	1.89	Upper 25%
*CCND3*	Protein	157	No	< 0.030	–	(28)	0.039^c^	2.32	Upper 25%
*CCND2*	Protein	57	No	0.042	–	(28)	0.047^f^	1.67	Lower 25%
*LGALS3*	mRNA	165	Yes	< 0.001	–	(29)	0.016^e^	0.61	Lower 25%
*USP28*	Protein	206	Yes	< 0.001	–	(30)	0.048^c^	0.29	Lower 30%
							0.014^d^	0.38	Lower 30%
*DIABLO*	Protein	84	No	< 0.050	–	(31)	0.239^g^	0.87	NA
*RB1*	Protein	311	No	0.030	–	(25)	0.898^g^	1.02	NA
*FGFR3*	mRNA	114	No	0.035	–	(32)	0.462^g^	0.92	NA
*CCND1*	Protein	157	No	< 0.020	–	(28)	0.997^g^	0.98	NA

a Significant P-value validated in a combined cohort (OS);

b Significant P-value validated in a combined cohort (DSS);

c, d, e, f Significant P-value validated in dataset GSE32548, GSE48075, TCGA, and GSE13507, respectively;

g *No significance P-value validated in any cohorts, “–” means no HR data, “NA” means not applicable*.

## Discussion

The discovery of prognostic biomarkers is a hot topic in translational research. In the current study, we present a convenient web server to assist researchers and clinicians to quickly screen and evaluate the prognostic value of genes in different cohorts of BC. As shown in a straightforward web interface, people without much bioinformatics experience can easily navigate OSblca to investigate genes of interests. In addition, users can perform survival analysis filtered by one or several factors according to the specific research purposes of their needs.

The validation of previously reported prognostic biomarkers in OSblac showed that our web tool is reliable and can be used in prognostic analysis for BC patients. Notably, 11 genes, such as *KPNA2* and *TP53*, were confirmed as prognostic biomarkers in the combined cohort, which indicated that these genes may be more widely applied as prognostic candidates for BC patients.

In summary, OSblca is a free online survival analysis web server that allows clinicians and researchers to rapidly analyze the prognostic value of a given gene in BC. We will keep updating OSblca to make it more powerful for the users.

## Data Availability

Publicly available datasets were analyzed in this study. This data can be found here: http://bioinfo.henu.edu.cn/BLCA/BLCAList.jsp.

## Author Contributions

GZ, QW, MY, and XG collected data, developed the server, and drafted the paper. QY, YD, XS, YA, and HD set up the server and performed the analyses. LX, WZ, and YW contributed to data analysis and paper writing. All authors edited and approved the final manuscript.

### Conflict of Interest Statement

The authors declare that the research was conducted in the absence of any commercial or financial relationships that could be construed as a potential conflict of interest.
